# Supplementary material for: Low-Level Endothelial TRAIL-Receptor Expression Obstructs the CNS-Delivery of Angiopep-2 Functionalised TRAIL-Receptor Agonists for the Treatment of Glioblastoma
Source: Molecules. 2021 Dec 14;26(24):7582. doi: 10.3390/molecules26247582 (PMC8706683; doi:10.3390/molecules26247582)
Supplement: Supplementary file 1 [file molecules-26-07582-s001.zip › molecules-1431043-supplementary.pdf]

Supporting Information for:

Low level endothelial TRAIL-receptor expression obstructs the CNS-delivery of Angiopep-2 functionalised TRAIL-receptor agonists for the treatment of Glioblastoma (GBM)

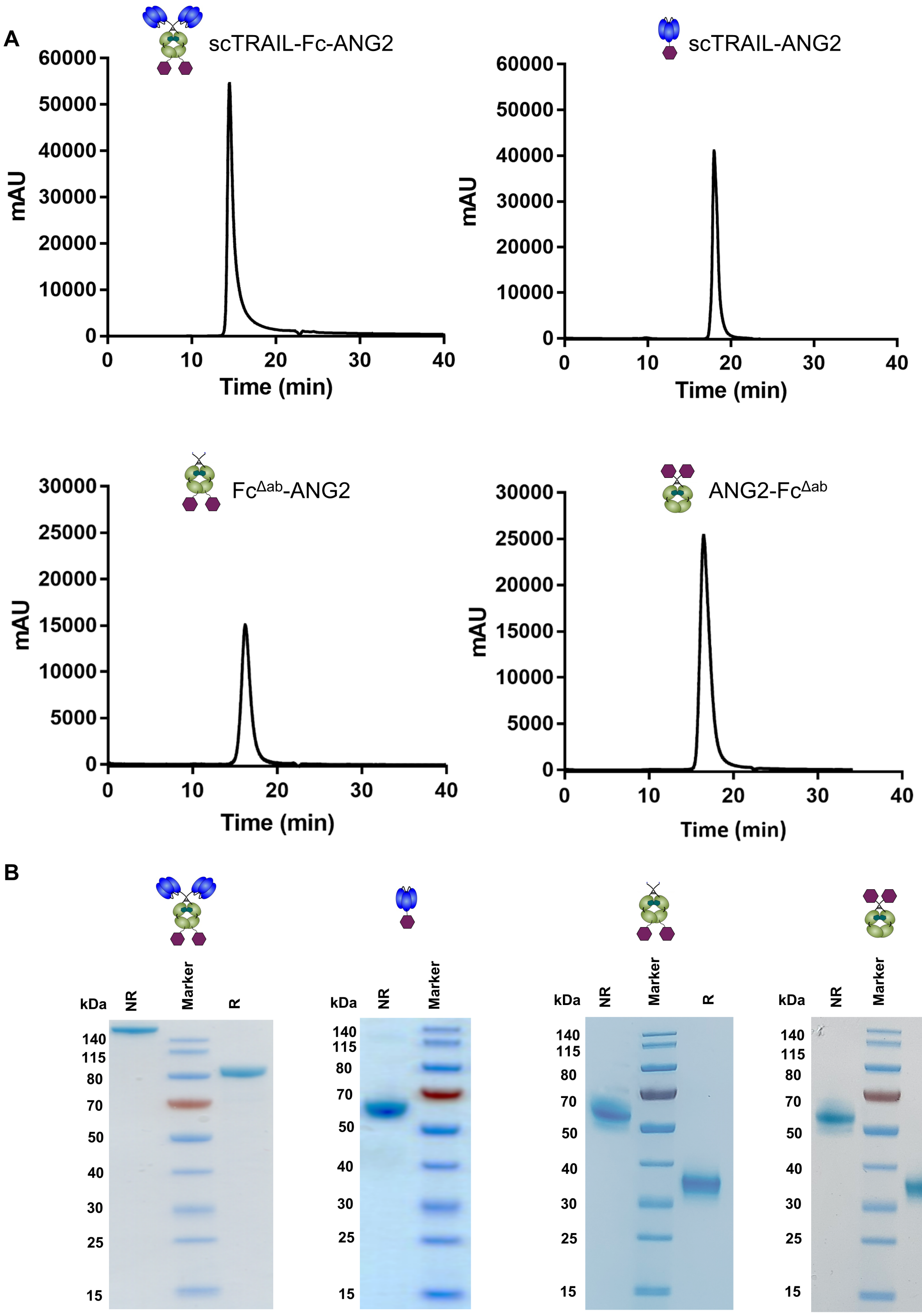

Figure S1: Biochemical characterization of the constructs. (A) Size exclusion HPLC chromatogram of indicated constructs with elution time. **(B)** SDS PAGE of indicated constructs was performed with (R) or without the reducing agent (NR) β-mercaptoethanol and stained with coomassie blue. 2 μg of the protein was loaded. kDa, molecular weight. mAU, milli absorbance unit.

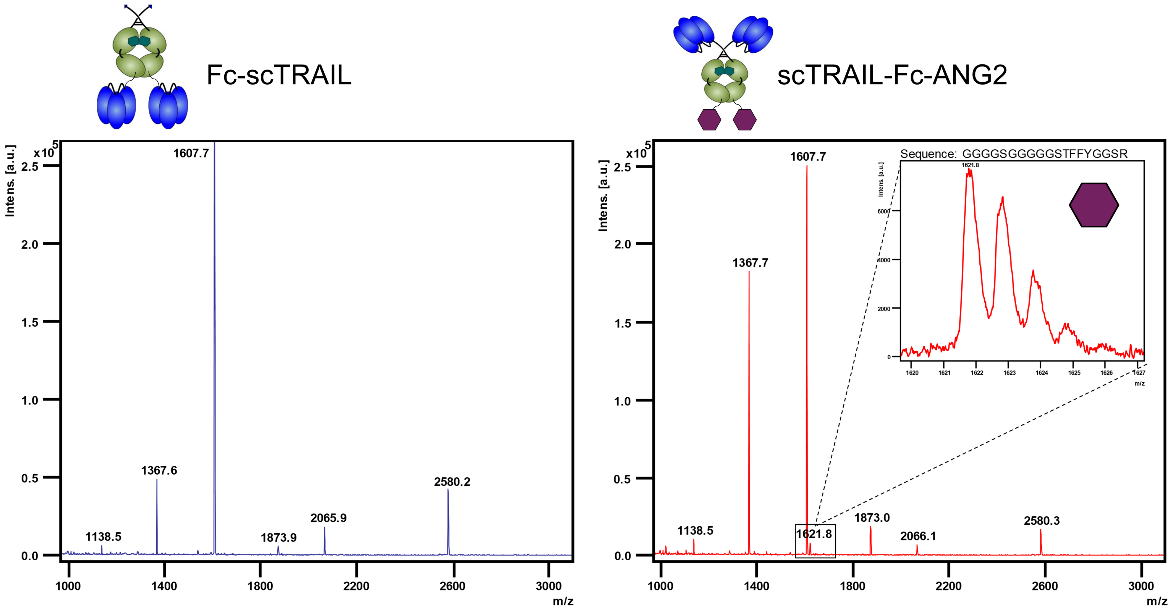

Figure S2: Verification of ANG2 presence by MALDI-TOF spectrometry. (A) MALDI-TOF spectra of Fc-scTRAIL (left) and scTRAIL-Fc-ANG2 (right) with magnification of spectra pertaining to ANG2 (insert).

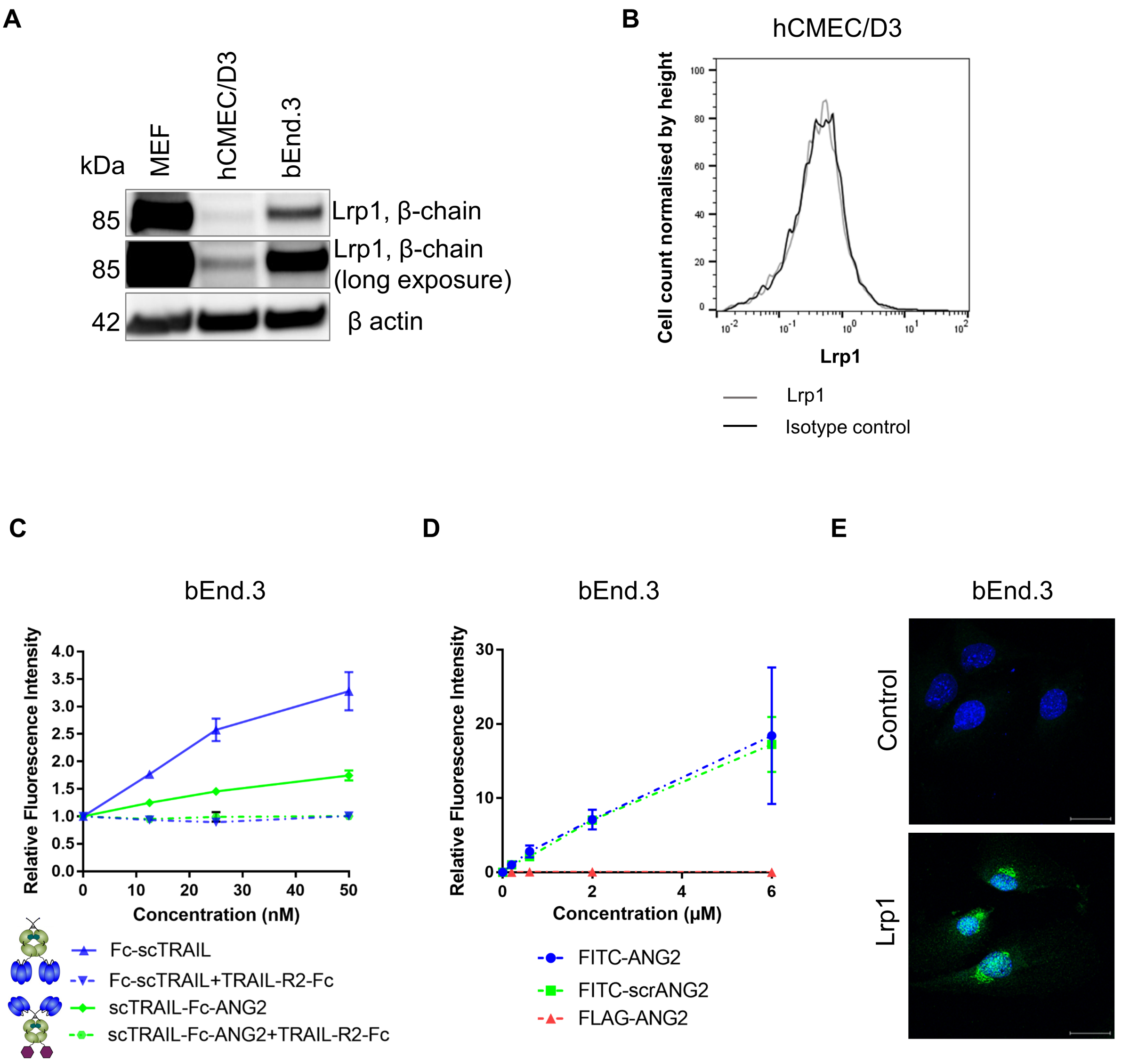

Figure S3: Binding of the constructs to the blood brain barrier cells is TRAIL-mediated. (A) Lysates of MEF, hCMEC/D3 and bEnd.3 cells were analysed for the expression of Lrp1 receptor by western blot analysis. β-Actin served as loading control. **(B)** The surface expression of Lrp1 on hCMEC/D3 cells was analysed by flow cytometry. Histograms are representative of three independent experiments. **(C)** bEnd.3 cells were incubated with indicated constructs for 1 h with or without 30 min preincubation of 100-fold molar excess of TRAIL-R2-Fc. Binding was assessed with anti-FLAG PE and flow cytometry. **(D)** Binding of bEnd.3 cells to FITC-labelled ANG2, scrambled ANG2 (scrANG2) or FLAG-tagged ANG2. Data points in C and D are mean ± SEM of three independent experiments. **(E)** Immunofluorescence staining of Lrp1 on bEnd.3 cells.

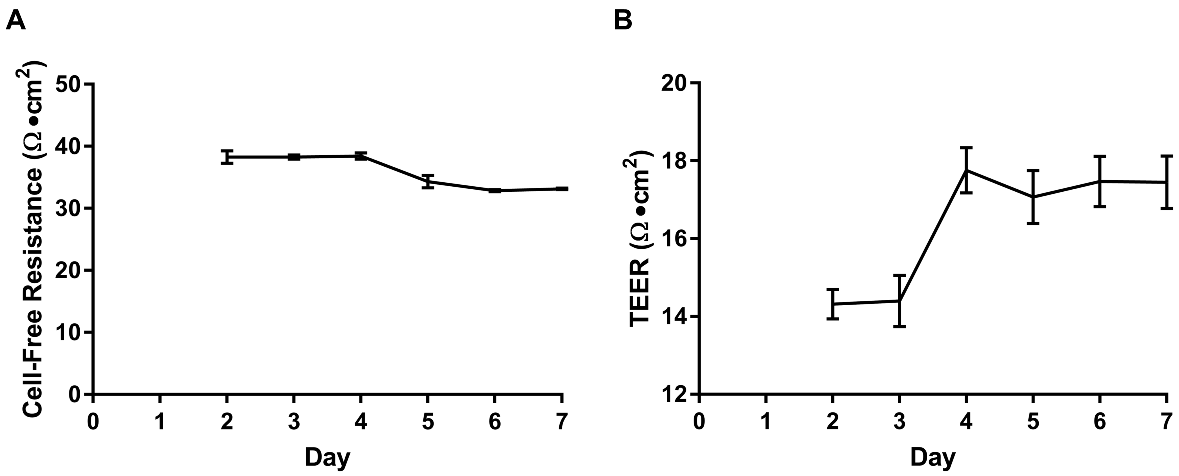

Figure S4: Transendothelial electrical resistance of b.End 3 transwell. (A) Cell free resistance of transwell insert for the transport assay (n=2) and **(B)** calculated TEER values of b.End3 subtracted by cell free resistance (n=16).
